# Association Between *CST3* Gene Polymorphisms and Large-Artery Atherosclerotic Stroke

**DOI:** 10.3389/fneur.2021.738148

**Published:** 2021-10-13

**Authors:** Yarong Ding, Zhe Xu, Yuesong Pan, Xia Meng, Xianglong Xiang, Hao Li, Liping Liu, Yongjun Wang

**Affiliations:** ^1^Department of Neurology, Beijing Tiantan Hospital, Capital Medical University, Beijing, China; ^2^China National Clinical Research Center for Neurological Diseases, Beijing, China; ^3^Center of Stroke, Beijing Institute for Brain Disorders, Beijing, China; ^4^Beijing Key Laboratory of Translational Medicine for Cerebrovascular Disease, Beijing, China

**Keywords:** cystatin C, polymorphism, biomarker, ischemic stroke, prognosis

## Abstract

**Objective:** Cystatin C, a marker of atherosclerosis, is encoded by *CST3*. We aimed to evaluate whether two single-nucleotide polymorphisms (SNPs) of *CST3* are correlated with large-artery atherosclerotic stroke (LAAS) and prognosis.

**Methods:** This subgroup analysis of the Third China National Stroke Registry (CNSR-III) enrolled acute ischemic stroke (AIS) patients within 7 days from August 2015 to March 2018 in China. rs13038305 and rs911119 of *CST3* were selected based on the strong association with cystatin C concentration.

**Results:** Two loci of *CST3* (rs13038305 and rs911119) were analyzed in 3,833 ischemic stroke patients. Carriers of T allele in rs13038305 and C allele in rs911119 tend to have lower serum cystatin C levels (*p* < 0.05). Compared with C/C as a reference in rs13038305, odds ratio (OR) of T/T was 0.486, 95% CI 0.237–0.994, *p* = 0.048. Per C allele of rs13038305 also showed an increased level of low-density lipoprotein cholesterol (LDL-C), β (95% CI) was 1.335 (1.008–1.250), *p* = 0.044. No correlation was found between the selected SNPs and stroke prognosis (functional outcome, recurrence, and mortality).

**Conclusions:** Carriers of the T allele in rs13038305 tend to have a lower proportion of LAAS. rs13038305 and rs911119 polymorphisms were likely to affect cystatin C concentration independently of kidney function.

## Introduction

Cystatin C (CysC, encoded by *CST3* on 20p11.21) is a candidate marker of glomerular filtration rate (1, 2). As a competitive inhibitor of cysteine proteases, CysC is also involved in the process of atherosclerotic plaque remodeling (3) and independently associated with cerebral artery stenosis and prognosis in stroke patients (4). The imbalance expression between CysC and cysteine proteases is a key factor of atherosclerosis. Although the effects of CysC on metabolic syndrome and atherosclerosis-related factors have been confirmed in observational studies (5), the underlying mechanism is still unclear (2, 6). The causal involvement or directionality of any causal effect on artery stenosis and ischemic stroke is yet to be proven (7).

Cerebrovascular disease is one of the leading causes of death in China, especially for ischemic stroke (IS) (8, 9). Intracranial and extracranial large-artery stenotic disease is one of the most common causes of ischemic stroke worldwide (10, 11). Large-artery atherosclerotic stroke (LAAS) was the most frequent subtype based on the Trial of Org 10 172 in Acute Stroke Treatment (TOAST) system (12), which was typically caused by intracranial artery stenosis (ICAS) and extracranial artery stenosis (ECAS). The stenosis of intracranial or extracranial arteries is also an important indicator of atherosclerosis. CysC has been proposed to participate in the process of atherosclerosis, thereby influencing the risk of cardiovascular disease (13). However, its role as a marker of atherosclerosis is still controversial. In this study, we aimed to assess whether two selected single-nucleotide polymorphisms (SNPs) of the *CST3* gene are correlated with LAAS and large-vessel stenosis as well as stroke prognosis.

## Methods

### Study Design and Subjects

This study was conducted based on the Third China National Stroke Registry (CNSR-III), a nationwide, multicenter, prospective registry study launched in China between August 2015 and March 2018, aiming to evaluate etiology, imaging, and biological markers of acute ischemic stroke (AIS). Detailed descriptions of the CNSR-III study have been reported previously (14). Blood samples were collected from 171 study sites for this prespecified biomarker subgroup analysis. We selected two SNPs of the *CST3* gene (rs13038305 and rs911119) as the robust association with the circulating concentration of CysC based on the previous study. Of note, these two SNPs were reported not associated with creatinine-based measures of renal function (15, 16). Finally, 3,833 subjects were included in our analysis. The protocol of the CNSR-III study was approved by the ethics committee of Beijing Tiantan Hospital.

### Data Collection and Blood Sample Test

Blood samples were collected on the first day of enrollment and transported through the cold chain to the central laboratory in Beijing Tiantan Hospital, where all serum specimens were stored at −80°C until testing was performed. Blood samples were sent to the laboratory to extract serum, plasma, and white blood cells and tested uniformly in the central laboratory of Beijing Tiantan Hospital according to the standardized methods.

The value of CysC was measured by the immunoturbidimetric method (Roche Cobas c501 analyzer with CysC assay); coefficient of variation (CV) of CysC was 2%. SNP analysis was accomplished by Kompetitive allele-specific polymerase chain reaction (KASP) method, which is also called allele-specific quantitative polymerase chain reaction (AQP)-based genotyping assay. To verify the accuracy of the KASP method and sample numbers, the quality controls required that the consistency between KASP and first-generation sequencing results in verified loci was >99%. The laboratory indices [including high-sensitivity C-reactive protein (hs-CRP), creatinine, high-density lipoprotein (HDL), low-density lipoprotein (LDL), triglyceride (TG), total cholesterol(TC), and creatinine] were tested by the central laboratory of Beijing Tiantan Hospital after admission. All measurements were performed by laboratory personnel blinded to the study status. Stroke subtypes are classified according to the TOAST system. The degree of stenosis was assessed according to computed tomographic angiography (CTA), magnetic resonance angiography (MRA) imaging, or conventional ultrasonography. More than 50% caliber reduction of the intracranial and extracranial artery was defined as ICAS and ECAS, respectively. To ensure the diagnosis standard was consistent, all images were independently evaluated by two trained neuroradiologists blinded to clinical information. A third neuroradiologist was involved for additional assessment if there was disagreement in certain cases.

### Outcome Assessment

The primary objective was to explore the impact of SNPs on LAAS and large-vessel stenosis. Additionally, we evaluated the association between individual SNPs and the poor functional outcome of modified Rankin scale (mRS 3–6) score, stroke recurrence, all-cause mortality, and the combined vascular events during 1 year of follow-up. The outcomes were obtained through clinic or telephone in 1-year follow-up. Assessment of endpoints was completed by trained research coordinators who were blinded to patients' baseline clinical information.

### Statistical Analysis

Haploview analysis software (http://www.broadinstitute.org/haploview) was used to perform Hardy–Weinberg equilibrium analysis. Pearson's χ^2^-test or Fisher's exact test was used for comparisons of LAAS and control group (indicating an etiological diagnosis of non-LAAS based on the TOAST system). Logistic regression and Cox regression analyses were performed to compute odds ratios (ORs) and hazard ratio (HR) with 95% confidence intervals (CIs) after adjusting the covariates to assess the correlation between *CST3* gene polymorphisms and outcomes in an addictive and dominant model. We also performed the ordinal logistic regression analysis between per major allele of the selected SNPs and atherosclerosis risk factors including CysC. Effect sizes (β) are presented as standard deviations of each trait per major allele to facilitate comparison between traits. Statistical analyses were performed using SAS software (version 9.4; SAS Institute, Cary, NC, USA). The level of significance was defined as *p* < 0.05 (two-sided).

## Results

### Baseline Characteristics

As a subgroup analysis of CNSR-III, two loci of the *CST3* gene (rs13038305 and rs911119) were analyzed in 3,833 ischemic stroke patients. Characteristics of enrolled patients compared with the entire CNSR-III cohort are shown in Supplementary Table 1. Patients enrolled in our analysis have more proportion of ischemic stroke, coronary artery disease, and higher National Institutes of Health Stroke Scale (NIHSS).

**Figure 1:**
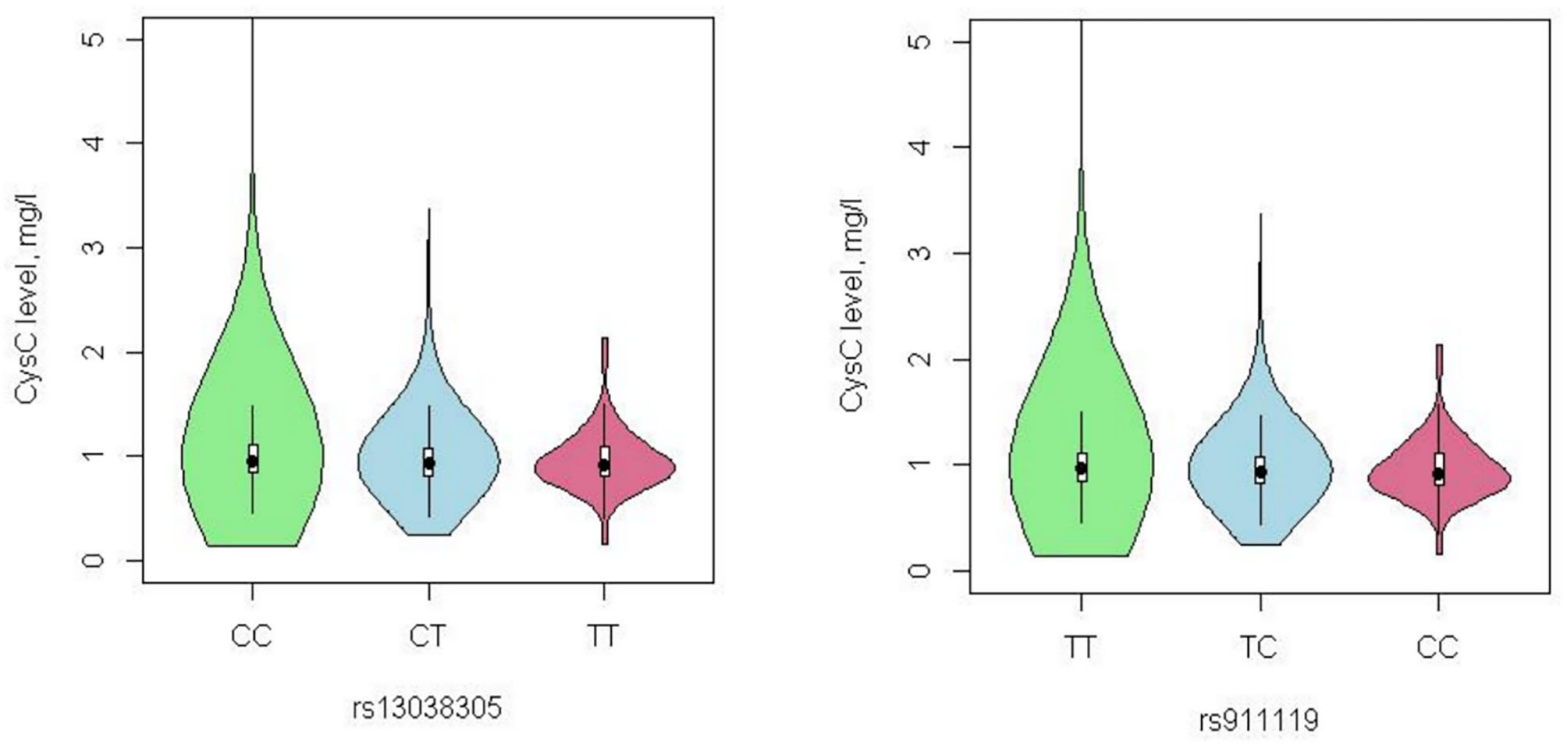
Cystatin C content in different genotype groups.

The distribution of alleles and genotypes of the two groups follows the Hardy–Weinberg equilibrium (rs13038305, *p* = 0.853; rs911119, *p* = 0.331). Figure 1 shows the organ diagram of CysC content in different genotype groups. Median CysC concentrations per genotype for rs13038305 were as follows: 1.012 mg/l (C/C), 0.990 mg/l (C/T), and 0.969 mg/l (T/T); for rs911119 were as follows: 1.014 mg/l (T/T), 0.988 mg/l (T/C), and 0.971 mg/l (C/C). Table 1 summarizes the clinical characteristics of enrolled LAAS patients and control subjects. There were no differences between the two groups in the clinical characteristic aspects of age, sex, body mass index (BMI), medical history of hyperlipidemia, smoking, alcohol drinking, creatinine-calculated estimated glomerular filtration rate (eGFRcr), and concomitant medication of antiplatelets and anticoagulants. NIHSS scores at admission are higher in the LAAS group (*p* < 0.001). Besides, the LAAS group showed more proportion of ischemic stroke, coronary artery disease, diabetes mellitus, and hypertension (*p* < 0.05).

**Table 1:** Characteristics of enrolled patients.

Characteristics^*^	**Total**	**LAAS group**	**Control group^†^**	***p*-value**
	**(*N* = 3,833)**	**(*N* = 1,005)**	**(*N* = 2,828)**	
Age, years, mean ± SD	62.8 ± 11.3	63.3 ± 11.2	62.6 ± 11.3	0.068
Male sex	2,602 (67.9)	697 (69.4)	1,905 (67.4)	0.246
NIHSS score at admission				<0.001
0–3	2,024 (52.8)	449 (44.7)	1,575 (55.7)	
≥4	1,809 (47.2)	556 (55.3)	1,253 (44.3)	
BMI	24.6 (22.7–26.7)	24.3 (22.5–26.7)	24.7 (22.8–26.7)	0.553
**Medical history**				
Ischemic stroke	904 (23.6)	267 (26.6)	637 (22.5)	0.010
Coronary artery disease	456 (11.9)	137 (13.6)	319 (11.3)	0.048
Hyperlipidemia	326 (8.5)	86 (8.6)	240 (8.5)	0.945
Diabetes mellitus	933 (24.3)	282 (28.1)	651 (23.0)	0.001
Hypertension	2,405 (62.7)	662 (65.9)	1,743 (61.6)	0.017
Smoking	1,193 (31.1)	323 (32.1)	870 (30.8)	0.419
Alcohol drinking	543 (14.2)	140 (13.9)	403 (14.3)	0.803
**Laboratory data**				
hs-CRP, mg/l	1.510 (0.700–4.420)	1.975 (0.805–5.410)	1.390 (0.670–4.040)	<0.001
CysC, mg/l	0.960 (0.850–1.100)	0.960 (0.848–1.100)	0.960 (0.850–1.100)	<0.001
TG, mmol/l	1.400 (1.060–1.900)	1.360 (1.070–1.800)	1.410 (1.060–1.920)	0.005
TC, mmol/l	3.980 (3.330–4.740)	3.940 (3.280–4.720)	3.990 (3.350–4.750)	<0.001
LDL-C, mmol/l	2.420 (1.830–3.130)	2.430 (1.840–3.110)	2.420 (1.830–3.130)	0.004
HDL-C, mmol/l	0.940 (0.760–1.130)	0.930 (0.770–1.111)	0.950 (0.760–1.140)	<0.001
eGFRcr, ml/min/1.73 m^2^	91.914 (79.770–100.972)	91.908 (80.546–100.591)	91.914 (79.622–101.00)	0.331
**Concomitant medication**				
Antiplatelets	3,733 (97.4)	982 (97.7)	2,751 (97.3)	0.458
Anticoagulants	399 (10.4)	101 (10.1)	298 (10.5)	0.664

*SD, standard deviation; LAAS, large-artery atherosclerosis stroke; CysC, cystatin C; NIHSS, National Institutes of Health Stroke Scale; BMI, body mass index; hs-CRP, high sensitivity C-reactive protein; TG, triglyceride; TC, total cholesterol; HDL, high-density lipoprotein; LDL, low-density lipoprotein; eGFRcr, creatinine-calculated estimated glomerular filtration rate*.

* *Variables were presented as median (interquartile range) or counts (percentages) unless otherwise indicated*.

† *Control group indicating etiological diagnosis of non-LAAS based on The Trial of Org 10 172 in Acute Stroke Treatment (TOAST) system*.

### Association Between Two Single-Nucleotide Polymorphisms of the *CST3* Gene and Large-Artery Atherosclerotic Stroke

The distributions of the alleles and genotypes were following Hardy–Weinberg equilibrium in both groups (rs13038305, χ^2^ = 0.317, *p* = 0.853; rs911119, χ^2^ = 2.206, *p* = 0.331). Table 1 shows that carriers of the T allele of the SNP rs13038305 tend to have a lower rate of LAAS. Compared with C/C as a reference, the *CST3* allelic frequency OR of T/T in the univariate analysis is 0.494, 95% CI 0.242–1.009, *p* = 0.053. After adjusting for covariate in multivariate analysis, adjusted OR was 0.486, 95% CI 0.237–0.994, *p* = 0.048. Carriers of the C allele of the SNP rs911119 tend to have a lower rate of LAAS. However, no statistically significant differences between genotypes and allele frequencies were found for SNP rs911119 between LAAS and control group (Table 2).

**Table 2:** Genotype and allelic frequencies of *CST3* SNPs in LAAS and control subjects.

**SNPs sites**		**LAAS group, *n* (%)**	Control group^*^, *n* (%)	**Univariate analysis**		**Multivariate analysis^†^**	
				**OR (95%CI)**	***p*-value**	**Adjusted OR (95% CI)**	***p*-value**
**rs 13038305**	C/C	778 (71.4)	2,135 (75.5)	Reference		Reference	
Genotypes	C/T	218 (21.7)	643 (22.7)	0.930 (0.782–1.107)	0.417	0.922 (0.774–1.099)	0.365
	T/T	9 (0.9)	50 (1.8)	0.494 (0.242–1.009)	0.053	0.486 (0.237–0.994)	0.048
	C/T+T/T	227 (22.6)	693 (24.5)	0.899 (0.758–1.066)	0.222	0.891 (0.750–1.057)	0.186
Allele	C	1,774 (88.3)	4,913 (86.9)	Reference			
	T	236 (11.7)	743 (13.1)	0.880 (0.752–1.028)	0.108	0.872 (0.746–1.020)	0.088
**rs 911119**	T/T	751 (74.7)	2,080 (73.6)	Reference		Reference	
Genotypes	C/T	244 (24.3)	695 (24.6)	0.972 (0.822–1.150)	0.744	0.961 (0.812–1.138)	0.648
	C/C	10 (1.0)	53 (1.9)	0.523 (0.265–1.032)	0.062	0.517 (0.261–1.023)	0.058
	C/T+C/C	254 (25.3)	748 (26.5)	0.940 (0.797–1.109)	0.4661	0.930 (0.788–1.098)	0.391
Allele	T	1,746 (86.9)	4,855 (85.8)	Reference		Reference	
	C	264 (13.1)	801 (14.2)	0.916 (0.789–1.064)	0.253	0.908 (0.781–1.055)	0.206

*SNPs, single-nucleotide polymorphisms; LAAS, large-artery atherosclerosis stroke; CI, confidence interval; OR, odds ratio*.

* *Control group indicating etiological diagnosis of non-LAAS based on The Trial of Org 10 172 in Acute Stroke Treatment (TOAST) system*.

† *Adjusted covariates including sex, age, smoking and drinking, hyperlipidemia, diabetes mellitus, hypertension, ischemic stroke, and coronary artery disease history*.

### Association Between Two Single-Nucleotide Polymorphisms of the *CST3* Gene and Intracranial Artery Stenosis or Extracranial Artery Stenosis

Table 3 shows the correlation between the two selected SNPs and large-artery stenosis. There was no association between the two SNPs and either ICAS or ECAS.

**Table 3:** Genotype and allelic frequencies of *CST3* SNPs in ICAS or ECAS group.

**SNP sites**		**ICAS or ECAS group, *n* (%)**	Control group^*^, *n* (%)	**Univariate analysis**		**Multivariate analysis^†^**	
				**OR (95% CI)**	***p*-value**	**Adjusted OR (95% CI)**	***p*-value**
**rs 13038305**	C/C	1,273 (76.2)	1,374 (76.1)	Reference		Reference	
Genotypes	C/T	366 (21.9)	406 (22.5)	1.028 (0.875–1.207)	0.738	1.012 (0.860–1.192)	0.882
	T/T	31 (1.9)	26 (1.4)	0.777 (0.459–1.316)	0.348	0.755 (0.443–1.286)	0.301
	C/T+T/T	1,273 (76.2)	1,374 (76.1)	1.008 (0.862–1.179)	0.919	0.992 (0.847–1.163)	0.921
Allele	C	2,912 (87.2)	3,154 (87.3)	Reference		Reference	
	T	428 (12.8)	458 (12.7)	0.988 (0.858–1.138)	0.867	0.973 (0.843–1.123)	0.709
**rs 911119**	T/T	1,237 (74.1)	1,338 (74.1)	Reference		Reference	
Genotypes	C/T	400 (24.0)	440 (24.4)	1.017 (0.870–1.189)	0.833	1.001 (0.855–1.173)	0.986
	C/C	33 (2.0)	28 (1.6)	0.784 (0.471–1.306)	0.350	0.768 (0.459–1.286)	0.316
	C/T+C/C	433 (25.9)	468 (25.9)	0.999 (0.858–1.163)	0.992	0.984 (0.843–1.147)	0.833
Allele	T	2,874 (86.1)	3,116 (86.3)	Reference		Reference	
	C	466 (14.0)	496 (13.7)	0.982 (0.857–1.125)	0.791	0.968 (0.843–1.111)	0.644

*SNPs, single-nucleotide polymorphisms; ICAS, intracranial artery stenosis; ECAS extracranial artery stenosis; CI, confidence interval; OR, odds ratio*.

* *Control group indicating patients without intracranial and extracranial artery stenoses*.

† *Adjusted covariates including sex, age, smoking and drinking, hyperlipidemia, diabetes mellitus, hypertension, ischemic stroke, and coronary artery disease history*.

### Genetic Variant and Cardiovascular Risk Factors

**Figure 2:**
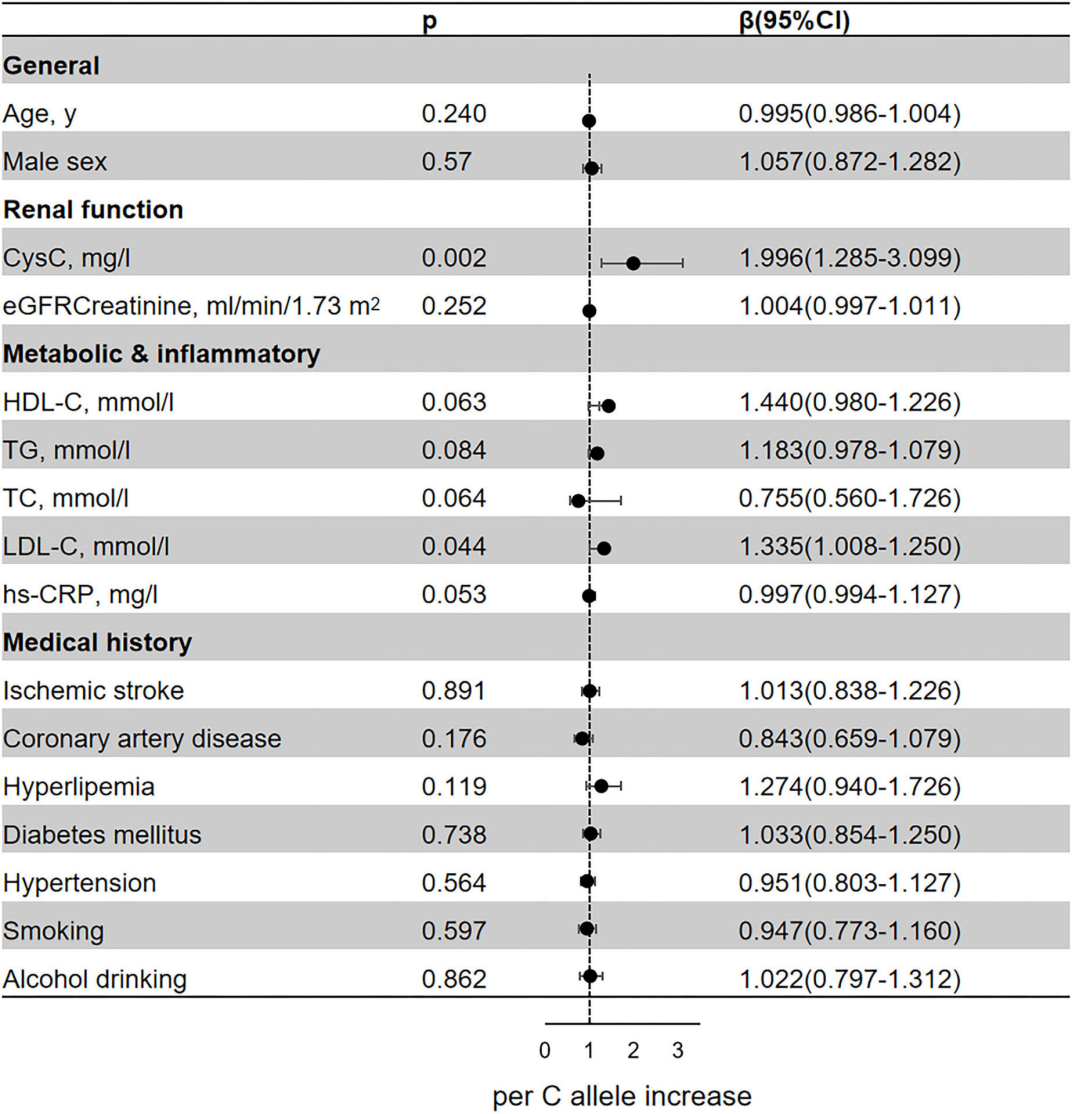
Association of the genetic variant (rs13038305 polymorphisms) with cardiovascular risk factors and traits. Effect sizes (β) are presented as standard deviations of each trait per major allele to facilitate comparison between traits. Adjusted for age, sex, CysC, eGFRcr, HDL-C, TG, TC, LDL-C, hs-CRP, smoking, alcohol drinking, ischemic stroke, coronary artery disease, hyperlipidemia, diabetes mellitus, and hypertension. BMI, body mass index; CysC, cystatin C; eGFRcr, creatinine-calculated estimated glomerular filtration rate; LDL, low-density lipoprotein; HDL, high-density lipoprotein; TG, triglyceride; TC, total cholesterol; hs-CRP, high-sensitivity C-reactive protein; CI, confidence interval.

**Figure 3:**
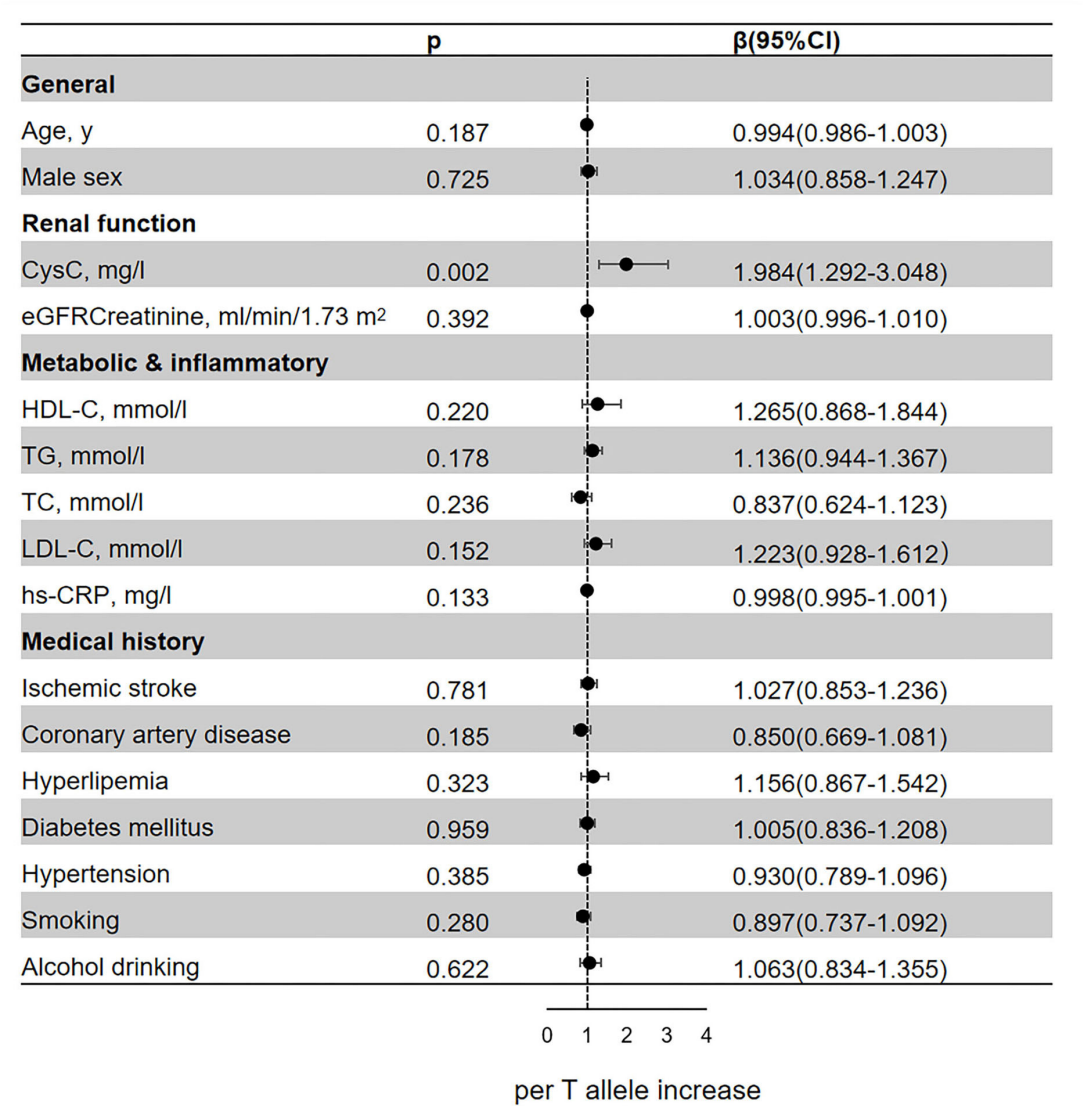
Association of the genetic variant (rs911119 polymorphisms) with cardiovascular risk factors and traits. Effect sizes (β) are presented as standard deviations of each trait per major allele to facilitate comparison between traits. Adjusted for age, sex, CysC, eGFRcr, HDL-C, TG, TC, LDL-C, hs-CRP, smoking, alcohol drinking, ischemic stroke, coronary artery disease, hyperlipidemia, diabetes mellitus, and hypertension. BMI, body mass index; CysC, cystatin C; eGFRcr, creatinine-based estimated glomerular filtration rate; LDL, low-density lipoprotein; HDL, high-density lipoprotein; TG triglyceride; TC, total cholesterol; hs-CRP, high-sensitivity C-reactive protein; CI, confidence interval.

In the regression analyses, rs13038305 and rs911119 were all related to the content of serum CysC (*p* < 0.05; Figures 2, 3). Of note, per C allele of rs13038305 also showed an increased level of LDL cholesterol (LDL-C), β was 1.335, and 95% CI 1.008–1.250 after adjustment [adjusted for age, sex, CysC, eGFRcr, HDL cholesterol (HDL-C), TG, TC, LDL-C, hs-CRP, smoking, alcohol drinking, ischemic stroke, coronary artery disease, hyperlipidemia, diabetes mellitus, and hypertension]. However, rs13038305 and rs911119 showed no significant association with other cardiovascular risk factors and traits in the increase of per major allele in the adjusted model. Besides, there is no correlation between these two SNPs and creatinine, but they are strongly correlated with the content of CysC.

### Clinical Outcomes in Different *CST3* Polymorphisms

We evaluated the risk prediction of genotype (rs13038305 and rs911119) for stroke prognosis during 1 year of follow-up (Table 4). No statistically significant association between the polymorphism rs13038305 or rs911119 and any outcome was observed.

**Table 4:** Clinical outcomes in different *CST3* polymorphisms.

**SNP sites**	**Outcomes**	**Genotypes**	**No. of events/total no. (%)**	Model 0^*^		**Model 1^†^**	
				**HR (95% CI)**	***p*-value**	**Adjusted HR (95% CI)**	***p*-value**
**rs13038305**	Poor functional outcome (mRS score 3–6)	C/C	362 (12.6)	–		–	
		T/C	118 (13.9)	1.118 (0.894–1.398)	0.327	1.104 (0.877–1.389)	0.399
		T/T	7 (11.9)	0.933 (0.420–2.069)	0.864	0.890 (0.395–2.005)	0.779
		C/T+T/T	125 (13.8)	1.106 (0.889–1.376)	0.817	1.089 (0.870–1.363)	0.456
	Stroke recurrence	C/C	279 (9.6)	(Reference)		(Reference)	
		T/C	84 (9.8)	1.018 (0.798–1.299)	0.887	1.016 (0.796–1.296)	0.901
		T/T	6 (10.2)	1.051 (0.468–2.359)	0.904	1.036 (0.461–2.325)	0.932
		C/T+T/T	90 (9.8)	1.020 (0.804–1.293)	0.871	1.017 (0.802–1.290)	0.890
	All-cause mortality	C/C	81 (2.8)	(Reference)		(Reference)	
		T/C	33 (3.8)	1.386 (0.925–2.078)	0.114	1.356 (0.905–2.033)	0.140
		T/T	2 (3.4)	1.212 (0.298–4.929)	0.788	1.178 (0.290–4.793)	0.819
		C/T+T/T	35 (3.8)	1.375 (0.925–2.044)	0.116	1.345 (0.904–1.999)	0.143
	Combined vascular events	C/C	292 (10.0)	–		–	
		T/C	87 (10.1)	1.008 (0.793–1.280)	0.951	1.005 (0.791–1.277)	0.967
		T/T	7 (11.9)	1.170 (0.553–2.476)	0.681	1.151 (0.544–2.436)	0.713
		C/T+T/T	94 (10.2)	1.018 (0.807–1.285)	0.879	1.015 (0.804–1.280)	0.902
**rs911119**	Poor functional outcome (mRS score 3–6)	T/T	351 (12.6)	–		–	
		T/C	128 (13.8)	1.112 (0.895–1.383)	0.338	1.102 (0.881–1.377)	0.396
		C/C	8 (12.7)	1.010 (0.477–2.138)	0.980	0.967 (0.450–2.081)	0.933
		C/T+C/C	136 (13.7)	1.106 (0.894–1.368)	0.355	1.093 (0.878–1.359)	0.426
	Stroke recurrence	T/T	270 (9.5)	–		–	
		T/C	93 (9.9)	1.036 (0.818–1.311)	0.771	1.033 (0.816–1.308)	0.786
		C/C	6 (9.5)	0.984 (0.438–2.209)	0.969	0.971 (0.432–2.180)	0.943
		C/T+C/C	99 (9.9)	1.032 (0.820–1.300)	0.787	1.029 (0.818–1.296)	0.806
	All-cause mortality	T/T	80 (2.8)	–		–	
		T/C	34 (3.6)	1.286 (0.861–1.921)	0.219	1.266 (0.848–1.892)	0.249
		C/C	2 (3.2)	1.115 (0.274–4.535)	0.879	1.081 (0.266–4.396)	0.914
		C/T+C/C	36 (3.6)	1.275 (0.861–1.890)	0.226	1.254 (0.846–1.859)	0.259
	Combined vascular events	T/T	283 (10.0)	–		–	
		T/C	96 (10.2)	1.020 (0.809–1.285)	0.868	1.017 (0.807–1.283)	0.883
		C/C	7 (11.1)	1.094 (0.517–2.316)	0.814	1.077 (0.509–2.281)	0.845
		C/T+C/C	103 (10.3)	1.025 (0.818–1.284)	0.832	1.021 (0.815–1.280)	0.855

*SNPs, single-nucleotide polymorphisms; CI, confidence interval; HR, hazard ratio; mRS, modified Rankin Scale*.

* *Model 0 was unadjusted*.

† *Model 1 was adjusted for age (in year, continuous) and sex (male or female)*.

## Discussion

Previous observational studies on CysC have confirmed that it was not simply a marker of renal function to affect cardiovascular and stroke outcomes (17–19). CysC might change metabolic status and promote the progress of metabolic disorders (20). Besides, since CysC is a potent competitive inhibitor of cysteine proteases (3) and involved in the progression of atherosclerosis, the balance cysteine protease and protease inhibitor (CysC) plays an important role in the pathogenesis of cerebral injury and functional rehabilitation (21). However, underlying mechanisms remain to be fully elucidated.

Our previous study has proven the relationship between CysC levels and poor functional outcome independent of renal function in 1 year after ischemic stroke (22). In this study, we selected two SNPs that have been reported to be strongly related to serum CysC level and tried to investigate the impact of SNPs on LAAS and large-vessel stenosis. We found that carriers of the T allele of SNP rs13038305 tend to have a lower proportion of LAAS. However, no correlation was found between the two SNPs and either ICAS or ECAS. The possible reason is that large-artery stenosis is not the only factor leading to LAAS, and our classification method to assess intracranial and extracranial large-vessel stenosis may miss some detailed information about the degree of vascular stenosis and the state of atherosclerotic plaque. T allele of the SNP rs13038305 tends to have a lower CysC level and higher CysC-based eGFR (eGFRcys) (6, 23). The minor allele of rs911119 was also associated with decreased serum CysC (2, 24). Consistent with previous research, we reported a strong association between the CysC concentration and the two SNPs. A lower CysC level may also indicate a lower risk of atherosclerosis and therefore a lower LAAS proportion. Besides, per major allele (C) of rs13038305 seems related to higher LDL-C concentration, which may cause changes in metabolic status. The observational study also found that the effect of CysC on prognosis might be modified by LDL-C level because of a potential interaction between lipids and CysC (25, 26). Our research indicated that CysC might be an important marker in determining subclinical atherosclerosis.

Although elevated CysC concentration was associated with cardiovascular risk factors and prognosis (17, 27, 28), Mendelian randomization studies based on the two selected SNPs revealed that genetic *CST3* seems not to be a causal risk factor of cardiovascular disease in the population-based prospective cohort study (2, 6, 29). In our study, we failed to find any association between these two SNPs and stroke outcome at 1 year. Previous studies have also confirmed that although the epidemiological relationship between plasma CysC level and coronary heart disease exists and is statistically independent of other risk factors such as atherosclerosis and renal function, there is insufficient genetic evidence. Even if the two selected SNPs were associated with increased CysC level, our study did not find that the genetic polymorphisms of these two loci resulted in impaired renal function, nor did it affect other risk factors that may cause impaired renal function. However, the specific functions of these two SNPs and the causal relationship between CysC concentration remain to be elucidated.

Although this subgroup analysis was based on the data from a large, multicenter, progressive cohort study, there were still several limitations. First, MRA, CTA, and ultrasonography are not the gold standards for assessing intracranial and extracranial stenosis; hierarchical evaluation of 50% instead of the detailed value of stenotic severity might be less accurate to some extent. Second, since there is a fluctuant level of inflammatory and metabolic markers, a one-time examination of plasma level might confound the mediator concentration. The concentration peak of biomarkers might be missed, and the variation could not be observed as well. However, even if we conducted plasma level examination for one time, the SNP frequency was consistent under normal circumstances. The variation of CysC levels might have less impact on the primary outcome. Third, the investigated cohort of ischemic stroke patients only enrolled a proportion of patients in China; thereby, the results are not necessarily applicable to a general population. The correlation between *CST3* and ischemic stroke needs further exploration.

## Conclusions

In conclusion, our study indicated that carriers of the T allele of the SNP rs13038305 tend to have a lower proportion of LAAS. rs13038308 and rs911119 polymorphisms were likely to affect CysC concentration independently of kidney function. Per major allele (C) of rs13038305 related to higher LDL-C concentration. However, no correlation was found between stroke prognosis and the polymorphisms at rs13038305 or rs911119.

## Data Availability Statement

The original contributions presented in the study are included in the article/Supplementary Material, further inquiries can be directed to the corresponding author/s.

## Ethics Statement

The studies involving human participants were reviewed and approved by the Ethics Committee of Beijing Tiantan Hospital. The patients/participants provided their written informed consent to participate in this study.

## Author Contributions

YW had full access to all the data in the study, takes responsibility for the integrity of the data and the accuracy of the data analysis, and designed and conceptualized this study. YD performed the experiments and drafted the manuscript. ZX interpreted the data. XM revised the article for intellectual content. HL interpreted the data and revised the manuscript. YP conducted the statistical analysis and interpreted the data. LL revised the article for intellectual content. XX conducted the statistical analysis. All authors contributed to the article and approved the submitted version.

## Funding

This work was supported by grants from the National Key R&D Program of China (2016YFC0901002 and 2018YFC1312903), National Science and Technology Major Project (2017ZX09304018), and Beijing Municipal Science & Technology Commission (D171100003017002 and Z181100001818001).

## Conflict of Interest

The authors declare that the research was conducted in the absence of any commercial or financial relationships that could be construed as a potential conflict of interest.

## Publisher's Note

All claims expressed in this article are solely those of the authors and do not necessarily represent those of their affiliated organizations, or those of the publisher, the editors and the reviewers. Any product that may be evaluated in this article, or claim that may be made by its manufacturer, is not guaranteed or endorsed by the publisher.
