# Supplementary material for: Association Between CST3 Gene Polymorphisms and Large-Artery Atherosclerotic Stroke
Source: Front Neurol. 2021 Oct 13;12:738148. doi: 10.3389/fneur.2021.738148 (PMC8548665; doi:10.3389/fneur.2021.738148)
Supplement: Supplementary file 1 [file Table_1.PDF]

Supplemental Material

Association between CST3 Gene polymorphisms and Large Artery Atherosclerotic Stroke

CONTENTS

Supplemental Table 1. Comparison between enrolled and excluded patients

Characteristics*	Enrolled (N=3833)	Excluded (N=11333)	P-value
Age, y, mean±SD	62.8±11.3	62.1±11.3	<0.001
Male, Sex	2602(67.9)	7762(68.5)	0.486
NIHSS at admission			0.017
0-3	2024(52.8)	6236(55.0)	
≥4	1809(47.2)	5097(45.0)	
BMI, mean±SD	24.8±3.4	24.7±3.3	0.021
Medical history			
Ischemic stroke	904(23.6)	2245(19.8)	<0.001
Coronary artery disease	456(11.9)	1152(10.2)	0.003
Hyperlipemia	326(8.5)	865(7.6)	0.083
Diabetes mellitus	933(24.3)	2577(22.7)	0.042
Hypertension	2405(62.7)	7089(62.6)	0.831
Smoking	1193(31.1)	3559(31.4)	0.747
Alcohol drinking	543(14.2)	1583(14.0)	0.760
Laboratory data			
hs-CRP, mg/l	1.5(0.7-4.4)	1.9(0.9-4.9)	<0.001
CysC, mg/l	1.0(0.9-1.1)	0.9(0.8-1.1)	<0.001
TG, mmol/l	1.4(1.1-1.9)	1.4(1.0-1.9)	0.001
TC, mmol/l	4.0(3.3-4.7)	4.0(3.3-4.7)	0.101
LDL-C, mmol/l	2.4(1.8-3.1)	2.3(1.7-2.9)	<0.001
HDL-C, mmol/l	0.9(0.8-1.1)	0.9(0.8-1.1)	0.415
eGFRcr, ml/min/1.73 m^2	91.9(79.8-101.0)	93.6(82.4-102.4)	<0.001
Concomitant medication			
Antiplatelets	3733(97.4)	10880(96.0)	<0.001
Anticoagulants	399(10.4)	1147(10.1)	0.610
1-year Outcomes			
mRS 3-6	487(12.9)	1494(13.6)	0.298
Stroke recurrence	369(9.6)	1104(9.7)	0.836
All-cause mortality	116(3.0)	382(3.4)	0.301
Combined vascular events	386(10.1)	1171(10.3)	0.644

Abbreviations： SD, standard deviation; NIHSS, National Institutes of Health Stroke Scale; BMI, Body mass index; CysC, Cystatin C; hs-CRP denotes high sensitivity C reactive protein; TG, triglycerides; TC, total cholesterol; HDL, high-density lipoprotein; LDL, low-density

lipoprotein; eGFRcr, creatinine-based estimated glomerular filtration rate.
mRS, modified Rankin Scale.
* Variables were presented as median (interquartile range) or counts (percentages) unless otherwise indicated.
